# Influence of curvature strain and Van der Waals force on the inter-layer vibration mode of WS_2_ nanotubes: A confocal micro-Raman spectroscopic study

**DOI:** 10.1038/srep33091

**Published:** 2016-09-13

**Authors:** Xiao Hu Wang, Chang Cheng Zheng, Ji Qiang Ning

**Affiliations:** 1 Department of Physics, HKU-Shenzhen Institute of Research and Innovation (HKU-SIRI), HKU-CAS Joint Laboratory on New Materials, The University of Hong Kong, Pokfulam Road, Hong Kong, China; 2 Mathematics and Physics Centre, Department of Mathematical Sciences, Xi’an Jiaotong-Liverpool University, Suzhou 215123, China; 3 State Key Laboratory of Functional Materials for Informatics, Shanghai Institute of Microsystem and Information Technology, Chinese Academy of Sciences, Shanghai 200050, China; 4 Vacuum Interconnected Nanotech Workstation, Suzhou Institute of Nano-tech and Nano-bionics, Chinese Academy of Sciences, Suzhou 215123, China

## Abstract

Transition-metal dichalcogenides (TMDs) nanostructures including nanotubes and monolayers have attracted great interests in materials science, chemistry to condensed matter physics. We present an interesting study of the vibration modes in multi-walled tungsten sulfide (WS_2_) nanotubes prepared via sulfurizing tungsten oxide (WO_3_) nanowires which are investigated by confocal micro-Raman spectroscopy. The inter-layer vibration mode of WS_2_ nanotubes, A_1g_, is found to be sensitive to the diameter and curvature strain, while the in-plane vibration mode, E^1^_2g_, is not. A_1g_ mode frequency shows a redshift by 2.5 cm^−1^ for the multi-layered nanotubes with small outer-diameters, which is an outcome of the competition between the Van der Waals force stiffening and the curvature strain softening. We also show that the Raman peak intensity ratio is significantly different between the 1–2 wall layered nanotubes and monolayer flat sheets.

Stimulated by the great success of carbon-based nanostructures including nanotubes, graphene and nanoparticles, transition-metal dichalcogenides (TMDs) nanostructures from nanotubes to monolayers have attracted a great deal of interest from fields, such as materials science, chemistry, and condensed matter physics in recent years^1^,^2^,^3^,^4^,^5^,^6^. For layered structures of TMDs, there have been fast developments in both understanding of fundamental properties and applications of electronic/optoelectronic devices, for example, energy band structure transition from indirect to direct bandgap when thinned to monolayers and remarkable layer-number dependent electronic and optical properties^7^,^8^,^9^,^10^,^11^. For TMD nanotubes, like carbon nanotubes, their electronic structures as well as physical and chemical properties also exhibit an interesting dependence on the structural properties such as chirality and diameter^12^,^13^. In particular, a recent theoretical study shows that Raman signals of the in-plane and out-of-plane (or inter-layer used in the present article) lattice vibration modes depend significantly and linearly on the strain in TMD nanotubes, and concludes that Raman spectroscopy is an excellent tool to determine the strain of the TMD nanotubes and hence monitor the progress of nanoelectromechanical experiments^14^. Meanwhile, due to their high stretchability, two-dimensional crystals demonstrate the potential in controlling their optical and electrical properties by means of strain engineering^15^, such as physically bending or elongating a flexible substrate^16^,^17^, piezoelectric stretching and thermal expansion of the substrate^18^,^19^, and controlled wrinkling^20^,^21^.

Thanks to its excellent nanomechanical behavior, WS_2_ nanotube has been used as an atomic force microscopic tip to probe deep grooves, which offers a substantial improvement to the imaging quality^22^. In the multi-walled TMD nanotubes, the curvature strain induced by bending stress and the inter-layer Van der Waals force shall jointly play a key role in determining their mechanical properties, which has been proved in carbon nanotubes^23^,^24^. To the best of our knowledge, however, there have been very few reports investigating the influence of curvature strain and Van der Waals force on the vibration modes of multi-walled WS_2_ nanotubes.

In this article, we attempt to fill in this void by controlling the sulfurization conditions and average diameter of the WS_2_ nanotubes. We firmly show that the inter-layer vibration mode (A_1g_) of multi-walled WS_2_ nanotubes is sensitive to the average diameter and wall layer number. The joint effects of curvature strain and Van der Waals force on the nanomechanical properties of multi-walled WS_2_ nanotubes can be precisely controlled to some extent.

## Results and Discussions

**Figure 1:**
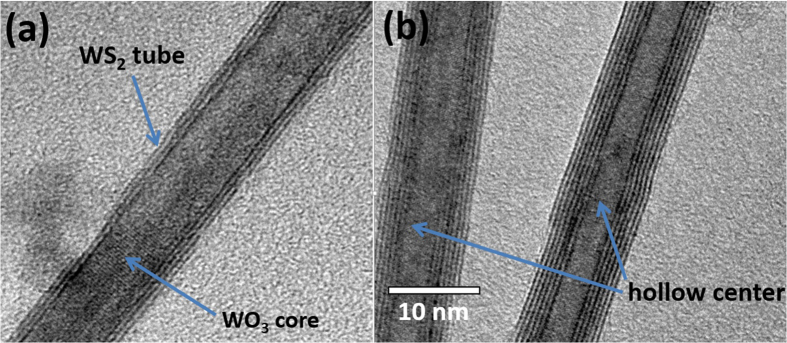
(**a**) HR-TEM image of a three layer walled WS_2_ nanotube that is partially filled by WO_3_; (**b**) HR-TEM image of a short piece of six layer walled nanotube.

**Figure 2:**
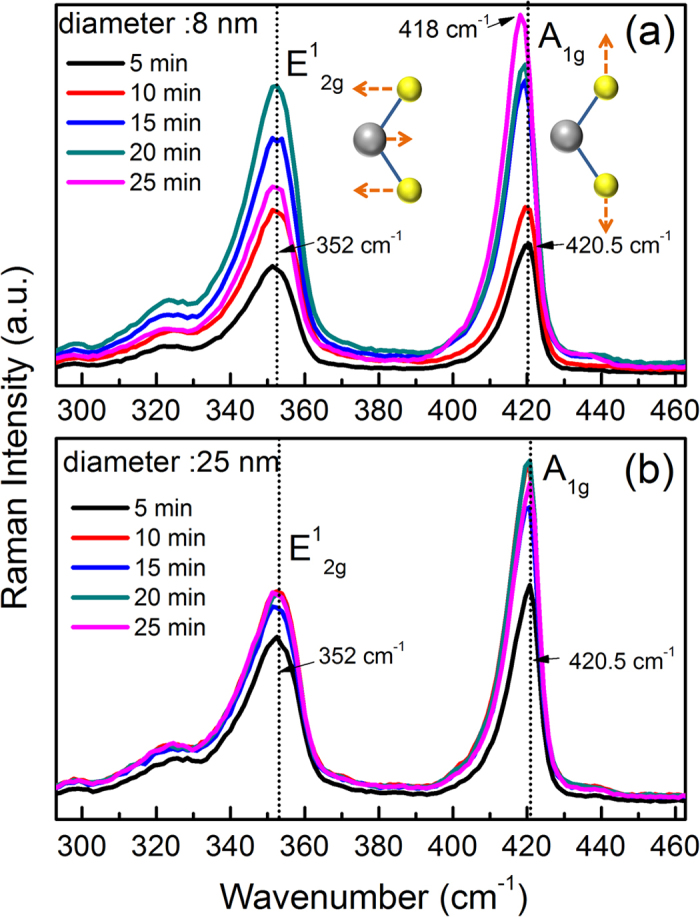
Micro-Raman spectra of WS_2_ nanotubes with different diameters: (**a**) 8 nm and (**b**) 25 nm. The sulfurization temperature was kept at 500 °C and the step interval of sulfurization time was lasted for 5 min.

Figure 1(a) shows the HR-TEM image of a three layer walled WS_2_ nanotube with outer diameter of ~10 nm, whereas Fig. 1(b) shows the HR-TEM image of a short piece of six layer walled nanotube. From the images shown in Fig. 1, one can see that the nanotubes have the similar outer diameters but different wall layer numbers. Both nanotubes are sulfurized from the same set of WO_3_ nanowires and temperature but the latter one with longer duration. It is interesting to see that the outer diameter remains the same while longer sulfurization increases the layer number from the inside. However, the inner core cannot be fully filled with WS_2_ nanotube due to the high strain when the diameter becomes smaller. Figure 2(a,b) depict the measured micro-Raman spectra of the WS_2_ nanotubes with the average diameters of 8 nm and 25 nm, respectively. The nanotubes were obtained via sulfurizing WO_3_ nanowires at 500 °C. In order to examine the dependence of characterized Raman modes of WS_2_ nanotubes on wall layer number and average diameter, two sets of nanotubes with the average diameters of 8 nm and 25 nm were prepared with different sulfurization durations of 5 min. interval under the same synthesis temperature of 500 °C, where the nanotube diameters are the minimum and maximum obtained from our sample preparation processes. At such temperature, it was found that the WO_3_ nanowires can be fully sulfurized within 25 min. During each 5 min. time interval, micro-Raman measurement was carried out on the sample. Although there are traces of WO_3_ core inside the nanotube for short sulfurization duration as shown in Fig. 1(a), the Raman spectra show no phonon mode related to W-O vibration. Meanwhile, when sulfurization time increases, the WO_3_ traces have disappeared as viewed from TEM images. And hence we conclude that the effect of WO_3_ is little in this Raman study. Similar to the cases of a few layers and monolayer WS_2_^25^, the Raman spectra of both set of nanotubes show the characteristic in-plane and inter-layer vibration mode of E^1^_2g_ and A_1g_. The Raman signal of both modes shows an increasing tendency as the sulfurization time increases, which is an indication that longer sulfurization duration results in higher degree sulfurization of WO_3_ nanowires, especially increase of wall layer number of resulted WS_2_ nanotubes. Interestingly, in sharp contrast to the case of monolayer WS_2_ sheet, the Raman scattering intensity of the inter-layer vibration mode A_1g_ is always stronger than that of the in-plane mode E^1^_2g_.

**Figure 3:**
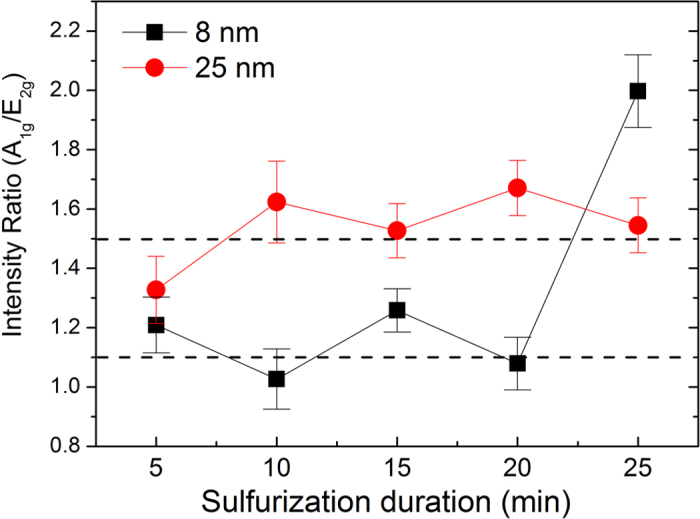
Raman peak intensity ratio (A_1g_/E^1^_2g_) of the two sets of WS_2_ nanotubes with different diameters versus the sulfurization time. Two dashed horizontal lines at 1.1 and 1.5 were drawn for guiding the eyes.

Moreover, Raman peak intensity ratio of A_1g_ and E^1^_2g_ shows some dependence on the average diameter, as shown in Fig. 3. For the nanotubes with the average diameter of 25 nm, the ratio kept at a constant of ~1.5 against the sulfurization time. For the nanotubes with the diameter of 8 nm, the ratio was ~1.1 for the sulfurization time ≤ 20 min. However, when the sulfurization time was up to 25 min., the ratio significantly increased to ~2.0. Such abnormal change in the Raman intensity ratio between the two characteristic modes is consistent with the substantial shift of the inter layer mode in frequency as argued below.

**Figure 4:**
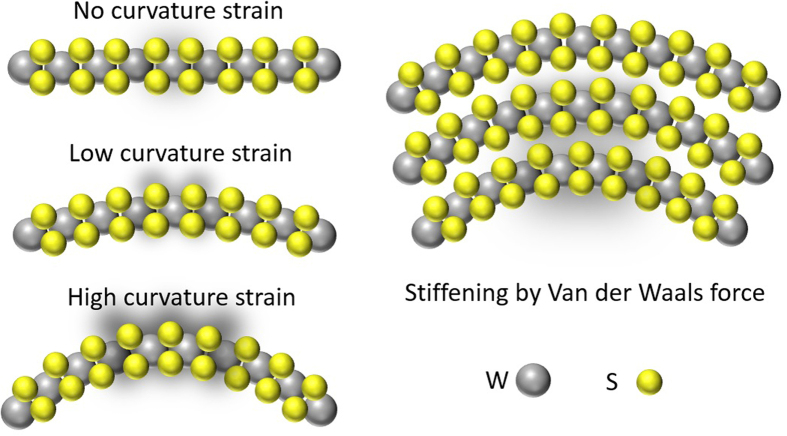
Schematic diagram of how the curvature strain and the Van der Waals force work. The left part shows an increase in curvature strain of monolayer WS_2_, which is denoted by an increase in the shaded area representing the vibration of the atoms. The right part illustrates the stiffening of lattice vibration due to inter-layer Van der Waals force for the curved WS_2_ multilayers.

In both sets of nanotubes, the peak position of the in-plane mode E^1^_2g_ was kept at 352.0 cm^−1^ and almost independent on the average diameter of the nanotubes and their wall layer numbers. For the inter-layer vibration mode A_1g_, the 8 nm nanotubes exhibit a red-shift in the peak position, i.e., moving from 420.5 cm^−1^ to 418.0 cm^−1^ when the sulfurization time was increased to 25 min. But for the 25 nm nanotubes, the peak still remains at 420.5 cm^−1^. The frequency shift of the inter-layer mode A_1g_ in the 8 nm nanotubes can be ascribed to a competition between the lattice strain caused by curvature and the inter-layer Van der Waals force. The curvature strain would lower the inter-layer vibration mode frequency while the Van der Waals force enhances. Similar to the two dimensional TMDs flat sheets, the increase of layer numbers would increase the Van der Waals force that stiffens atom vibration making the A_1g_ mode to blueshift^26^,^27^. On the other hand, the curvature strain is characterized by the curvature or strain energy^23^ defined as the energy difference for atoms in the nanotube compared with that of a flat plane. As obtained from the density-functional-based tight binding calculations^12^,^13^,^23^, the strain energy is inversely proportional to the square of the nanotube’s diameter and becomes negligible for diameters larger than 6 nm for the case of WS_2_^13^. Therefore, for the 25 nm nanotubes, the curvature strain is insignificant and only the stiffening of Van der Waals force may play a dominant role in determining the frequency of the inter-layer mode. That is why the peak position of A_1g_ is almost unchanged like the bulk case. However, for the 8 nm nanotubes, with increasing the sulfurization time, the wall layer number may increase and simultaneously the inner diameter of nanotube may get smaller and smaller. Eventually, the curvature energy becomes dominant over the Van der Waals force, causing a redshift of 2.5 cm^−1^ for A_1g_ mode, as shown in Fig. 2(a). Figure 4 shows a schematic diagram of how the curvature strain and the Van der Waals force work.

**Figure 5:**
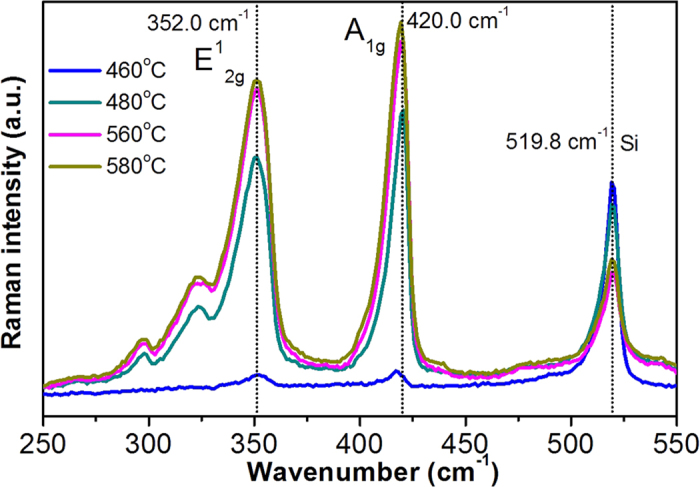
Micro-Raman spectra of the nanotubes with diameter of 10 nm for different sulfurization temperatures.

In order to further test the idea, we can find a medium case in which the Van der Waals force gradually becomes dominant. Figure 5 shows micro-Raman spectra of WO_3_ nanowires with average diameter of 10 nm sulfurized at different temperatures from 460 to 580 °C and at each temperature for 5 min. sulfurization. At 460 °C, both the vibration modes of WS_2_ start to appear while below this temperature both modes were absent. As the sulfurization temperature further increases, these two modes get stronger and eventually their Raman spectral features, such as line shape, relative intensity and peak positions, are in good agreement with those shown in Fig. 2. Therefore, 460 °C was a critical temperature for sulfurizing WO_3_ nanowires into WS_2_ nanotubes. At this critical temperature, WS_2_ nanotubes are believed to have only one or two wall layers. Note that the Raman peak of the inter-layer mode was located at 418.0 cm^−1^ and the intensity is quite weak. As expected, the Van der Waals force stiffening effect was observed for the 10 nm WS_2_ nanotubes with increasing the sulfurization time. For example, the inter-layer mode blueshifts towards 420.5 cm^−1^ eventually.

**Figure 6:**
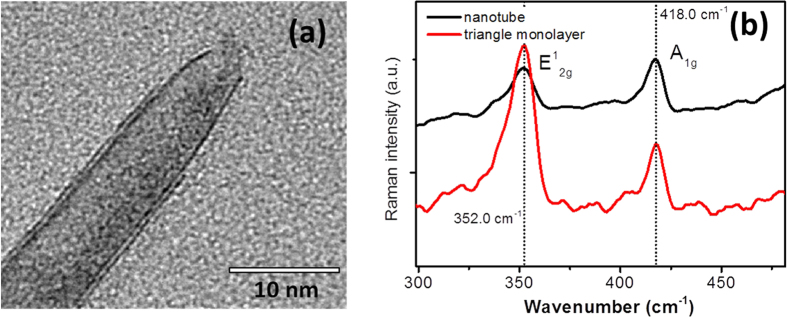
(**a**) HR-TEM image of a WS_2_ nanotube with only one or two wall layers; (**b**) Comparison of micro-Raman spectra between the 10 nm nanotubes prepared at the sulfurization temperature of 460 °C and a triangle shaped monolayer flake grown with CVD.

At last, we would like to make a comparison between the Raman spectrum of the WS_2_ nanotubes obtained at the sulfurization temperature of 460 °C and a triangle shaped monolayer flake prepared with chemical vapor deposition (CVD)^25^. Strain engineering on flat monolayer TMDs to modify the band structure has been a hot topic where many experimental progresses has been achieved^15^. The strong photoluminescence (PL) in monolayer MoS_2_ has demonstrated spectral shift with either uniaxial or biaxial strain^18^,^28^. Theoretical calculations predict that further increasing the strain can transform energy band from direct to indirect gap^29^. Different from synthesizing WS_2_ nanotube in this study, present experimental techniques on strain engineering are achieved by gradually curving or elongating the flat monolayer through various methods. Thus, it is interesting to compare properties of nanotube with that of strain induced monolayers. Figure 6(a) shows HR-TEM image of an individual WS_2_ nanotube with only one or two wall layers, whereas (b) depicts the micro-Raman spectra of the 10 nm nanotubes and a triangle shaped monolayer flake. However, unlike the strong PL for triangle monolayer, we have not detected any PL signal from the 1–2 walled WS_2_ nanotube indicating either tensile strain induces the energy band transition or the band structure for double walled nanotube is already indirect gap regardless of the level of the strain. According to previous works^27^, the identification criteria for monolayer 2D WS_2_ are not solely dependent on the frequency shift of the two characteristic modes, but also the intensity ratio between the two modes^25^. The latter criterion could be more important. From the Raman spectra of Fig. 6(b), it can be seen that the two vibration modes show almost identical peak positions: the in-plane mode E^1^_2g_ at 352.0 cm^−1^ and the inter-layer mode A_1g_ at 418.0 cm^−1^. However, the intensity ratio between the two modes is very different for the nanotube and monolayer flat sheet. The ratio is about 1.1 for the nanotubes with one or two wall layers, and is much smaller than 1 for the monolayer flat sheets. Again, our results show that the Raman intensity ratio between the two characteristic modes could be a more important criterion for identifying layer number of 2D WS_2_ flat sheets.

To conclude, a systematic investigation of micro-Raman scattering was conducted on WS_2_ nanotubes prepared via sulfurizing WO_3_ nanowires under different conditions. We firmly show that the inter-layer mode A_1g_ of WS_2_ nanotubes with small diameters can be significantly affected by the competition between the Van der Waals forces and curvature strain. And for nanotubes with 1–2 wall layers, their Raman spectrum closely resembles to that of two-dimensional monolayer flakes, but the intensity ratio differs largely.

## Methods

The growth of WS_2_ nanotubes was carried out in a horizontal tube furnace with high purity tungsten oxide (WO_3_) nanowires and sulfur powder as the preliminary reactants. WO_3_ nanowires were synthesized on silicon (100) wafer by thermal evaporating tungsten trioxide powder, and the average diameter of the nanowires can be changed from 6 nm to tens of nanometer via changing the source temperature during growth^30^. The WO_3_ nanowires were sulfurized at different temperatures ranging from 460 °C to 580 °C and each temperature for the same sulfurization duration (5 min.), or at the same temperature but for different durations. The control over the average diameter of the WS_2_ nanotube was achieved by synthesizing WO_3_ nanowires with desired diameter as the starting material. The wall layer number of the nanotubes was controlled by adjusting the sulfurization time and temperature. It should be noted that an oxygen deficient condition was kept in the furnace during the sulfurization process. High-resolution transmission electron microscopic (HR-TEM) observation of individual WS_2_ nanotubes was performed on FEI Tecnai G2 20 S-TWIN TEM (FEI, USA). Raman characterization of the samples was carried out on a WITec Alpha confocal micro-Raman system. The 514.5 nm laser line from a Melles Griot Ar+ gas laser was employed as the excitation source in the confocal micro-Raman spectroscopic measurements^25^.

## Additional Information

**How to cite this article**: Wang, X. H. *et al.* Influence of curvature strain and Van der Waals force on the inter-layer vibration mode of WS_2_ nanotubes: A confocal micro-Raman spectroscopic study. *Sci. Rep.* **6**, 33091; doi: 10.1038/srep33091 (2016).
